# Mindfulness-based stress reduction training supplemented with physiological signals from smartwatch improves mindfulness and reduces stress, but not anxiety and depression

**DOI:** 10.1371/journal.pone.0322413

**Published:** 2025-04-23

**Authors:** Sylwia Sumińska, Andrzej Rynkiewicz

**Affiliations:** 1 Department of Ergonomics, Central Institute for Labour Protection - National Research Institute, Warsaw, Poland; 2 Faculty of Psychology, University of Warsaw, Warsaw, Poland; Qatar University College of Nursing, QATAR

## Abstract

**Introduction:**

Mindfulness-Based Stress Reduction (MBSR) helps counteract the negative consequences of stress. An essential aspect of mind-body therapies is learning to be mindful of emotional reactions and bodily sensations, a process defined as interoceptive awareness. This awareness can also be enhanced by providing physiological feedback from a smartwatch. However, the impact of using smartwatch-generated physiological signals during mindfulness training has not been studied yet. The study aims at verifying, whether physiological signals from a smartwatch would support the MBSR.

**Methods:**

We conducted a mixed-design randomized controlled trial to investigate the effects of MBSR training, with and without monitoring physiological signals via a smartwatch, on mental functioning parameters, with measurements taken at baseline and after 8 weeks. Participants were classified into three groups (N = 72): the MBSR group, the MBSR + smartwatch group, and the control group. Between measurement sessions, two groups of participants were engaged in MBSR training, while the third group did not participate in any training.

**Results:**

Results showed a significant reduction in subjectively perceived stress levels, eating disorder symptoms, and intrusive ruminations in both groups participating in MBSR, compared to the control group. However, a notable difference emerged between the two MBSR groups: in the group with smartwatches, a significant increase in mindfulness was observed. In contrast, in the MBSR group without smartwatches, there was a significant decrease across multiple stress-related components, including: anxiety, cognitive impairment, addictions, sleep disorders symptoms, behaviors indicating lack of entertainment, and poor functioning.

**Conclusions:**

The results suggest that supplementing MBSR with monitoring interoceptive signals by a smartwatch enhances mindfulness, and maintains the effect of stress and eating disorders symptoms reduction but does not decrease anxiety nor improve general mental functioning. This imposes the need for further research to investigate mechanisms involved when observing interoceptive signals by a smartwatch.

## Introduction

Stress is the body’s natural response to the challenges it faces. This response occurs in both physical and psychological demands, encompassing physiological, emotional, and behavioral reactions [1–3]. According to the World Health Organization, stress is defined as a state of worry or mental tension triggered by a difficult situation. It leads to difficulties in relaxation and is associated with a broad spectrum of negative emotions, including anxiety and irritability. Prolonged high stress plays a key role in developing mental and physical health problems, e.g., depression [4], anxiety disorders [5], sleep disturbances [6,7], and an increased risk of using psychoactive substances, including alcohol [8,9], among other issues.

It seems that the main pathogenic mechanism of chronic stress is associated with sustained excessive arousal [10], often characterized by anxiety [11]. That may lead to the development of neurotic and depressive episodes. Chronic stress is rooted in difficulties initiating appropriate coping mechanisms to manage this heightened arousal. The self-regulation mechanism governs the regulation of emotions, thoughts, attention, and behavior in specific situations enabling effective responses to environmental demands [12–14]. Chronic self-regulatory failures increase the risk of developing psychopathology conditions [15–17]. For instance, chronic perceived failure in self-regulating negative emotions poses a risk for depression development [18–20]. Moreover, ineffective self-regulation is linked to impulsivity in attention deficit hyperactivity disorder (ADHD) [21], a tendency to ruminate in depressive disorders [22,23], emotion dysregulation in anxiety disorder [24,25], and physical health troubles, financial decision-making, and engaging in criminal behaviors [26].

One way to improve self-regulation is through the development of mindfulness. Mindfulness is a form of awareness characterized by attentively experiencing the present moment in a non-judgmental manner [27]. The essence of mindfulness lies in openness, curiosity, and acceptance of the arising experiences, combined with a nonreactive orientation. Such experiences encompass thoughts, emotional reactions, body sensations, and noticing stimuli coming from the environment [28]. The state of mindfulness is the opposite state of common daily mental activities, which are mind wandering [29], operating on autopilot [30], or suppressing unwanted experiences [31]. Enhanced awareness and mindfulness of one’s thoughts, feelings, and bodily sensations lead to a reduction in automatic and unreflective behaviors. This awareness fosters the recognition of mind wandering, enabling individuals to refocus on tasks. In addition, it promotes greater acceptance of unpleasant emotions and thoughts, discouraging their suppression. Self-regulation consistent with the idea of mindfulness entails directing attention to one’s present moment experiences in an accepting and non-judgmental manner. This attitude promotes emotional and behavioral regulation conducive to psychological well-being [27]. Many mindfulness concepts suggest that self-regulation, among other factors, is a central mechanism responsible for beneficial changes resulting from increased mindfulness. Self-regulation is included in the framework of Vago and Silbersweig [32], alongside mechanisms such as self-awareness and self-transcendence. Improving self-regulation involves enhancing several of its aspects, including attention regulation, body awareness, emotion regulation, and shifts in self-perception [18,33]. Similarly, Tang et al. highlighted that mindfulness promotes effective self-regulation through mechanisms such as effective emotion regulation, attention control, and self-awareness [34]. Additionally, Schuman-Olivier et al. expanded this framework by incorporating motivation and learning processes as crucial contributors to self-regulatory improvement [35]. Researchers consistently emphasize the aspect of emotion regulation [36,37], as well as body awareness [38] as significant for maintaining good mental health.

Activation of mindfulness, along with the associated emotional, cognitive, and motivational processes, is not straightforward and requires appropriate training and practice. One of the examples of learning mindfulness is participation in Mindfulness-Based Stress Reduction (MBSR). It is a type of training that was developed for patients with stress-related conditions and chronic pain [39]. MBSR aims to teach participants to become mindful of their emotional reactions and bodily sensations through guided practices (e.g., meditation, body scan, mindful yoga, gentle stretching) and by incorporating mindfulness into everyday life. MBSR lasts eight weeks and includes weekly two-and-a-half-hour group sessions led by a certified trainer, along with daily guided mindfulness practice [40]. It helps treat somatic diseases, including cardiovascular diseases, skin conditions, and mental disorders, including anxiety disorders, depression, chronic pain, substance abuse, and eating disorders [41–45]. Through training, individuals experience an increase in vitality, a decrease in perceived stress and stress-related symptoms, and an improvement in overall quality of life. There is also observed change in emotional reactivity and behavioral regulation.

Learning to be mindful of emotional reactions and bodily sensations is a critical process for mind-body therapies such as mindfulness-based interventions [46–49]. The perception of bodily sensations is generally termed interoception [50]. It has been demonstrated that the positive impact of mindfulness on depressive symptoms was mediated by interoceptive awareness, which contributed to an increased ability to avoid becoming distracted by unpleasant experiences [47] and a capacity for decentration [49]. Cultivating mindfulness of one’s sensations and bodily experiences contributes to the growth of interoceptive awareness [50,51]. Interoceptive awareness allows individuals to recognize physiological processes associated with affective states [52,53]. This awareness is particularly valuable in stressful situations where it facilitates the recognition and regulation of emotions [52–56]. Noticing and understanding bodily sensations plays a crucial role in human functioning [33,50,56–58]. Conversely, deficits in interoceptive awareness are a characteristic feature of emotion disorders [53,59,60]. Furthermore, interoceptive ability is weakened due to chronic stress [61].

Interoceptive awareness can also be supported by providing physiological signals from the body through electronic equipment. Studies have shown that immediate feedback on heartbeats enhances interoceptive awareness related to cardiac functioning [62]. Similarly, monitoring blood pressure with commercial smartwatches can contribute to a drop in blood pressure, primarily by fostering greater awareness of blood pressure levels and promoting self-monitoring [63]. Notably, an 8-week mindfulness program targeted at individuals with elevated blood pressure, which incorporated blood pressure monitoring, resulted in improved interoceptive awareness, better adherence to dietary recommendations, and significant nutritional changes [64]. Furthermore, emerging research suggests that smart devices can play a significant role in stress management [65–68]. Participants in a 4-week mindfulness-based intervention who used a device to assess respiratory patterns reported a greater decrease in stress, tension, and anxiety compared to a waitlist control group [68]. Similarly, integrating breathing techniques with dedicated brain-sensing devices has been associated with enhanced declines in stress, anxiety, and mental fatigue compared to the intervention alone [69]. Many studies reveal that smart devices can aid in forming healthy lifestyle habits, promoting physical activity, managing stress, and improving quality of life [70–73]. Smart devices can detect stress, cognitive load, or relaxation state based on heart rate, skin conductance, and accelerometers [66,74–76]. It is also possible to identify stress by analyzing heart rate variability, cortisol level, or body temperature [77].

Feedback from smart devices, such as smartwatches, can enhance interoceptive awareness by monitoring bodily sensations, thereby supporting effective self-regulation. Therefore, a stress-focused mind-body intervention such as MBSR may benefit from incorporating interoceptive signals provided by a smartwatch. Real-time observation of physiological signals and stress level monitoring through automatic feedback enables the identification of emotionally challenging situations as well as the body’s reactions to them. Smartwatch users can more quickly recognize stress-inducing situations and build their self-efficacy in effectively regulating emotions [51,65,67]. Research indicates that continuous and non-invasive health monitoring supports the development of new coping strategies [78]. Despite the potential inherent in monitoring information generated by smartwatches regarding our physiological parameters and its increasing popularity in health-related research, it has not yet been investigated whether it can help enhance body awareness and self-regulation in MBSR participants. Recent studies have explored the use of applications integrated with smart devices that provide guided breathing exercises or meditation as a means of emotion regulation [66,68,69]. To our best knowledge, our study is the first to complement MBSR with additional physiological parameter monitoring via a smartwatch. Unlike studies relying on pre-installed meditation programs in smart devices, our participants applied mindfulness principles learned through MBSR. We hypothesized that notifications about high stress levels would enable participants to identify emotionally challenging situations and subsequently encourage them to directly engage in mindfulness practice. Moreover, tracking physiological responses to stress in real time may provide users with valuable insights into their body’s reactions, further enhancing their ability to regulate emotions effectively. In light of the growing prevalence of smartwatches, there is a need to assess their usability and potential for application in various contexts.

The study aimed to investigate whether engaging in MBSR training would reduce perceived stress and improve mental health. Additionally, it examined whether continuous monitoring of physiological parameters via a smartwatch throughout the 8-week MBSR program could amplify the effects of mindfulness by influencing self-regulation abilities and interoceptive awareness. We hypothesized that the 8-week MBSR training program would significantly impact participants’ ability to cope with negative symptoms associated with functioning in adverse environmental conditions, such as those related to their work. Specifically, we expected that among individuals who had previously experienced the consequences of excessivestress but began attending training sessions regularly, the severity of these symptoms would decrease, and indicators of mental health would improve. This effect was anticipated to be even stronger in the group whose participants, in addition to attending MBSR sessions, regularly monitored their physiological parameters using a smartwatch. The reference point for the expected differences described above was the results of identical measurements obtained from the control group whose participants were not engaged in the MBSR training.

## Materials and methods

### Participants

A total of 215 individuals signed up for the study by completing an initial survey posted on social media. Participants were informed of the opportunity to partake in the complimentary MBSR training, contingent upon their consent to participate in the measurement of subjective stress levels and general mental functioning and physiological responses to induced stress, the results of which are not presented in this article. The recruitment process began on August 16, 2021, and ended on February 23, 2022. Despite significant interest in the project, a considerable number of individuals did not meet the eligibility criteria for the study. Only individuals who declared high level of stress over the past month were invited. Eligibility for the study was based on receipt of a score at or above six sten (upper limit of average scores and high scores) on the PSS-10 questionnaire. Other inclusion criteria based on the survey were: age 25–40, being healthy with no chronic diseases, or neurological and psychiatric disorders (e.g., hypertension, diabetes, cardiovascular disease, epilepsy, depression, anxiety disorders, substance use disorder, psychosomatic disorder, chronic pain), not taking medications that could affect the central nervous system (e.g., antidepressants, antianxiety drugs, sleeping pills), not participating in the psychotherapy, and no prior experience with MBSR. These criteria were verified through a prepared questionnaire. Inclusion criteria were established to eliminate confounders that could affect physiological parameters (not analysed in this article) e.g., age or chronic diseases that may affect physiological processes. In addition, all eligible participants underwent an initial interview conducted by a certified MBSR trainer, who assessed potential contraindications to participation in the training.

G*Power software determined the sample size [79]. A medium effect size (f = 0.25), alpha value of 0.05, and power of 0.80 were used to calculate a minimum total sample size of 42 participants. To account for a potential high dropout rate during the MBSR program, the number of participants was increased to ensure that at least the recommended 42 individuals completed the study. Ultimately, 57 female and 15 male subjects participated in the study. Subjects were between the ages of 25–40 (*M* = 29.3; *SD* = 3.83). As was assumed, most individuals received high scores (above seven sten) on the PSS-10 questionnaire.

Subjects were randomly assigned to two groups participating in MBSR training and one control group according to a simple randomization procedure with a 1:1:1 allocation. Due to the decision of some participants to withdraw from the control group or MBSR participation because of unavailability on the scheduled session dates, a portion of the initially qualified individuals was, upon providing consent, included in the control group. Sessions for both MBSR groups were conducted by the same MBSR trainer on previously scheduled and communicated dates. The control group received treatment as usual without participating in MBSR. All participants received compensation for their participation in the study. We decided to establish a control group that would not participate in the training to ensure that the observed changes in the MBSR groups are a result of the training itself rather than the natural dynamics of change over time. Additionally, a treatment-as-usual control condition reduces the risk of overestimating the effect size [80], which is supported by the observed smaller improvement in this condition compared to the waitlist condition All persons who participated in the required number of MBSR sessions (two absences were allowed) were included in the analyses. There were also individuals who resigned from the MBSR training due to the excessive time commitment it required or due to contracting COVID-19.

The study was carried out by three independent researchers. One researcher was responsible for recruiting participants, assigning them to one of the three groups, and providing them with smartwatches and diaries The second researcher was responsible for conducting measurements according to the standard procedure for all participants, regardless of their group assignment, and did not possess detailed knowledge of the tested hypothesis. Similarly, the third researcher - the certified MBSR trainer had no information about the study procedure and conducted MBSR training following the standard recommendations. The emerging potential differences were verified using appropriate statistical analyses, i.e., checking intergroup differences during the initial measurement.

The study was conducted according to the guidelines of the Declaration of Helsinki, and approved by the Ethics Committee of Cardinal Stefan Wyszynski University in Warsaw. Written informed consent was obtained from all subjects involved in the study. In the case of participants using smartwatches, written informed consent was obtained for data registration by a smartwatch and the use of the data for research purposes. Respondents were assured of anonymity and confidentiality of the data.

### Procedure

**Fig 1:**
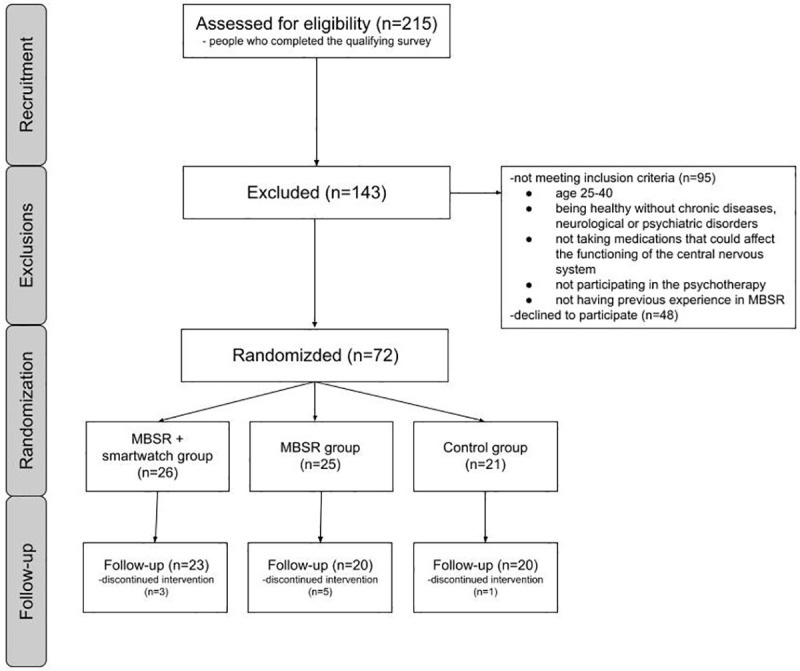
The flow diagram summarizing participant recruitment, exclusions, and randomization.

At the beginning of the initial measurement, the baseline level of stress and mental health parameters were assessed for each participant. Then, participants were assigned to three groups: a) group with MBSR training and monitoring physiological parameters via smartwatch (*N* = 26), b) group with MBSR training (*N* = 25), c) control group (treatment as usual) without training (*N* = 21). During the training phase, which lasted two months, the size of groups was reduced because some participants resigned. Finally, 23, 20, and 20 individuals remained in the groups, respectively (Fig 1). These individuals took part in a final measurement of stress and mental health parameters.

MBSR training was implemented according to a standard procedure and based on the authorized curriculum guide [40]. It lasted eight weeks and included weekly two-and-a-half-hour sessions and one six-hour silent session. The training consisted of independent exercises based on formal practice (e.g., Body Scan, Gentle Hatha Yoga, Sitting Meditation, Walking Meditation), informal practice (mindfulness in daily life exercises, i.e., awareness of breath, pleasant and unpleasant events), and a theoretical introduction. A certified MBSR trainer with experience in leading MBSR groups conducted the training. Training sessions were held in a stationary manner in the afternoons in fixed groups of ten individuals. Participants were asked to practice at home by guided audio recordings (30–50 minutes daily). Two absences were allowed in the group sessions.

The MBSR group with monitoring physiological parameters was requested to wear smartwatches 24/7. Monitoring bodily functions was performed using an advanced commercial smartwatch, the Garmin Venu SQ (Garmin, Ltd; Olathe, KS, USA), purchased before the study. This smartwatch model continuously measures some biological indicators, e.g., heart rate, breath frequency, and body movements (accelerometer). Based on these parameters, devices provide estimated information about all-day stress leve, sleep quality, and Body Battery^TM^. All this information is visible on the 1.3” LCD. The choice of model was motivated by its popularity. Participants were requested to check the results that appear in the smartwatch application twice a day (at the same time of day) and then record them in diaries prepared before the study. In particular, participants recorded information on the average stress level for the whole day, duration of rest, length of sleep, stages of sleep, average heart rate, number of steps, and Body Battery. This information did not contribute data of significant physiological value, therefore the data collected by the smartwatches were not analyzed. Smartwatches were used only to test the hypothesis that quasi-physiological feedback affects mental health while participating in MBSR by improving interoceptive awareness.

During the initial session, participants were provided with smartwatches. Settings tailored to each participant’s gender and body type were configured. The functionalities of the smartwatch were explained, and participants were instructed to monitor the relevant parameters using the provided diaries. They were informed that the smartwatch provides real-time stress level data and sends notifications when stress levels are elevated. This information aims to assist participants in identifying stressful situations and eliciting a stress response, while also encouraging them to take appropriate action to reduce stress. Participants were asked to use these moments as opportunities to pause, observe their experiences mindfully, in accordance with the principles of mindfulness that would be taught during the MBSR program.

### Stress and well-being measures

Cohen’s Perceived Stress Scale, PSS-10 [81]. We used a Polish version of the scale adapted by Juczyński and Ogińska-Bulik [82]. The scale enables the assessment of the intensity of stress related to own life situation over the past month. Eligibility for the study was based on scores on the PSS-10 questionnaire. The test has satisfactory psychometric properties in terms of relevance and reliability, i.e., the Cronbach alpha index was 0.86. For the study sample, Cronbach’s alpha in the initial measurement was 0.85.

Depression Anxiety and Stress Scale, DASS-21 [83]. It is a screening tool for assessing the intensity of anxiety, depressive symptoms, and stress level over the past week. The scale was used to check changes after 8 weeks. Due to the absence of a validated Polish version of the scale during the study period, the scale was translated using the back-translation method for research purposes. The structure and the three subscales show good internal consistency, convergent validity, and discriminant validity [83–85]. For the study sample, Cronbach’s alpha in the initial measurement was 0.81 for stress, 0.83 for anxiety, 0.90 for depression and in the final measurement was 0.84 for stress, 0.79 for anxiety, 0.87 for depression.

General Functioning Questionnaire, GFQ-58 [86]. It is a Polish-language screening tool for general functioning and severity of psychopathological symptoms in the past seven days. The scale was used to check changes after 8 weeks. The psychometric properties of the scale are satisfactory with Cronbach’s alpha of 0.89–0.92. The authors recommend using the subscales of the questionnaire rather than interpreting the total score. For this study, the results of the following scales were analyzed: poor functioning at work and home (*α* = 0.32–0.67), lack of entertainment (*α* = 0.65–0.82), poor social relationships (*α* = 0.63–0.79), cognitive impairments (*α* = 0.76–0.88), addictions (*α* = 0.77–0.80), depressive symptoms (*α* = 0.62–0.86), anxiety symptoms (*α* = 0.82–0.87), eating disorder symptoms (*α* = 0.45–0.55), sleep problems (*α* = 0.86–0.87), and somatic symptoms (*α* = 0.69–0.76). For the study sample, Cronbach’s alpha in the initial measurement was 0.61 for poor functioning, 0.73 for lack of entertainment, 0.64 for poor social relationships, 0.80 for cognitive impairments, 0.75 for addictions, 0.72 for depressive symptoms, 0.84 for anxiety symptoms, 0.16 for eating disorders symptoms, 0.88 for sleep problems, 0.44 for somatic symptoms and in the final measurement was 0.64 for poor functioning, 0.73 for lack of entertainment, 0.62 for poor social relationships, 0.78 for cognitive impairments, 0.71 for addictions, 0.77 for depressive symptoms, 0.78 for anxiety symptoms, 0.36 for eating disorders symptoms, 0.86 for sleep problems, 0.63 for somatic symptoms.

Mindful Attention Awareness Scale, MAAS [27]. We used a Polish version of this scale adapted by Radoń [87]. The MAAS is used to measure the trait of mindfulness, that is, the tendency to activate a specific state of attention resulting from continuously directing it to what is happening in the present moment in a non-evaluating and non-judgmental way. The scale was used to check changes after 8 weeks. The psychometric properties of the scale are satisfactory with Cronbach’s alpha of 0.81–0.85. For the study sample, Cronbach’s alpha in the initial measurement was 0.89 and in the final measurement was 0.87.

Event Related Rumination Inventory, ERRI [88]. We used a Polish version of this scale adapted by Ogińska-Bulik and Juczyński [89]. ERRI scores are treated as responses to a specific negative life event and consist of intrusive rumination and deliberate rumination. The scale was used to check changes after 8 weeks. The Polish version of ERRI has good psychometric properties with Cronbach’s alpha of 0.96 for intrusive rumination and 0.92 for deliberate rumination. For this study, we analyzed intrusive rumination. For the study sample, Cronbach’s alpha for intrusive rumination in the initial measurement was 0.93 and in the final measurement was 0.96.

## Results

We used the Shapiro-Wilk test to check the normality of the distributions of initial and final measurements. Since most of the variables did not meet the normality criterion, non-parametric tests were employed for their analysis. In the remaining cases, parametric test analyses were performed. The study investigated potential group differences in variable severity levels at baseline and post-intervention using either one-way ANOVA or the Kruskal-Wallis test (Table 1). Furthermore, the impact of MBSR training on the study variables was assessed through paired t-tests or Wilcoxon tests to determine any changes over time. The analyses were carried out in the IBM SPSS Statistics 27.

**Table 1:** Means, standard deviations, and p-values of DASS-21, MAAS, ERRI, and GFQ-58 scales at the initial and final measurement sessions in all groups.

	Measurement	MBSR+smartwatch	MBSR	Control group		
		*M* ± *SD*	*M* ± *SD*	*M* ± *SD*
**DASS-21 Stress**	Initial	15.91 ± 7.65	17.30 ± 9.37	19.50 ± 7.59	n.s*	
	Final	10.87^a^ ± 8.52	13.26 ± 6.64	16.70^b^ ± 6.72	*p* = 0.014*	*eta*^*2*^ = 0.10
		*p* = 0.01*	*p* **= 0.046**	n.s		
		*d* Cohena = 0.63	*d* Cohena = 0.49			
**DASS-21 Anxiety**	Initial	7.45 ± 7.15	9.20 ± 9.60	11.10 ± 7.53	n.s*	
	Final	4.26 ± 4.01	5.79 ± 5.85	8.50 ± 7.67	n.s*	
		n.s*	*p* = 0.049*	n.s*		
			*d* Cohena = 0.50			
**DASS-21 Depression**	Initial	11.73 ± 8.45	13.40 ± 9.70	13.80 ± 8.53	n.s*	
	Final	8.96 ± 7.95	10.63 ± 6.96	10.70 ± 7.63	n.s*	
		n.s*	n.s	n.s		
**MASS**	Initial	53.13 ± 9.80	55.55 ± 13.26	54.61 ± 12.13	n.s	
	Final	59.57 ± 10.34	59.40 ± 12.10	57.11 ± 10.67	n.s	
		*p* **= 0.004**	n.s	n.s		
		*d* Cohena = -0.68				
**GFQ-58 poor functioning**	Initial	2.99 ± 0.77	2.89 ± 0.74	3.04 ± 0.57	n.s*	
	Final	2.78 ± 0.66	2.55 ± 0.71	2.73 ± 0.60	n.s*	
		n.s*	*p* **= 0.027**	*p* **= 0.007**		
			*d* Cohena = 0.54	*d* Cohena = 0.68		
**GFQ-58 lack of entertainment**	Initial	2.96 ± 0.67	2.96 ± 0.70	3.16 ± 0.90	n.s*	
	Final	2.72 ± 0.76	2.53 ± 0.51	2.99 ± 0.89	n.s*	
		n.s	*p* = 0.01*	n.s		
			*d* Cohena = 0.69			
**GFQ-58 poor social relationships**	Initial	2.17 ± 0.82	2.14 ± 0.66	2.16 ± 0.66	n.s*	
	Final	1.86 ± 0.67	1.95 ± 0.59	2.09 ± 0.61	n.s*	
		n.s*	n.s	n.s		
**GFQ-58 cognitive impairments**	Initial	2.71 ± 1.04	2.97 ± 1.00	3.07 ± 0.68	n.s*	
	Final	2.54 ± 0.72	2.43 ± 0.93	2.78 ± 0.77	n.s*	
		n.s	*p* = 0.017*	n.s		
			*d* Cohena = 0.65			
**GFQ-58 addictions**	Initial	1.32 ± 0.46	1.61 ± 0.58	1.44 ± 0.53	n.s*	
	Final	1.23 ± 0.31	1.15 ± 0.33	1.41 ± 0.49	n.s*	
		n.s*	*p* = 0.004*	n.s*		
			*d* Cohena = 0.80			
**GFQ-58 depressive symptoms**	Initial	2.15 ± 0.67	2.28 ± 0.83	2.20 ± 0.48	n.s*	
	Final	2.03 ± 0.73	2.00 ± 0.66	1.96 ± 0.53	n.s*	
		n.s*	n.s	n.s		
**GFQ-58 anxiety symptoms**	Initial	1.91 ± 0.62	2.15 ± 0.73	2.25 ± 0.67	n.s*	
	Final	1.76 ± 0.64	1.71 ± 0.49	2.08 ± 0.52	n.s*	
		n.s*	*p* **= 0.021**	n.s*		
			*d* Cohena = 0.56			
**GFQ-58 eating disorder symptoms**	Initial	1.74 ± 0.50	1.82 ± 0.74	1.90 ± 0.56	n.s*	
	Final	1.43^a^ ± 0.48	1.43 ± 0.39	1.80^b^ ± 0.53	*p* = 0.035*	*eta*^*2*^ *= 0.12*
		*p* = 0.039*	*p* = 0.015*	n.s		
		*d* Cohena = 0.47	*d* Cohena = 0.59			
**GFQ-58 sleep problems**	Initial	1.88^a^ ± 0.84	2.50 ± 1.06	2.65^b^ ± 1.11	*p* = 0.030*	*eta*^*2*^ *= 0.11*
	Final	1.82 ± 0.73	1.87^a^ ± 1.08	2.31^b^ ± 0.78	*p* = 0.032*	*eta*^*2*^ *= 0.06*
		n.s*	*p* = 0.011*	n.s		
			*d* Cohena = 0.58			
**GFQ-58 somatic symptoms**	Initial	1.92 ± 0.64	1.94 ± 0.64	1.98 ± 0.53	n.s*	
	Final	1.67 ± 0.50	1.89 ± 0.60	1.89 ± 0.60	n.s*	
		n.s	n.s*	n.s*		
**ERRI intrusive rumination**	Initial	21.96 ± 5.52	18.30^a^ ± 6.44	23.65^b^ ± 6.04	*p* **= 0.019**	*eta*^*2*^ *= 0.12*
	Final	18.43 ± 7.17	13.75^a^ ± 7.36	21.20^b^ ± 7.84	*p* **= 0.008**	*eta*^*2*^ *= 0.15*
		*p* **= 0.048**	*p* **= 0.01**	n.s*		
		*d* Cohena = 0.44	*d* Cohena = 0.64			

The table shows results for comparisons between groups (in rows) and between the initial and final measurements (in columns) with p-value and effect size.

a, b Results of pairwise comparisons between group means are shown by letters - means with different letters in the same row are significantly different (p<.05, Bonferroni). Analyses were carried out using parametric and non-parametric tests according to the distribution of results of the variables.

*Analysis performed using non-parametric tests.

### Baseline levels

The analyses revealed that the groups differed in the level of variables only in the case of two variables during the baseline measurement, i.e., GFQ-58 sleep problems (*χ*^2^(2) = 7.03, *p* = 0.03) and ERRI intrusive ruminations (*χ*^2^(2) = 7.89, *p* = 0.019). Post hoc analysis revealed that the MBSR+smartwatch group reported a lower level of sleep problems than the control group (*p* = 0.03) and the MBSR group reported a lower level of intrusive ruminations than the control group (*p* = 0.019). These effects could potentially influence the measurements of changes relative to the baseline, as described by the Law of Initial Value [90]. But in both cases, we observed higher baseline levels of parameters in control groups, and decreasing of them in experimental groups was expected. Therefore, the postulated direction and magnitude of changes were opposite to possible artifacts. For all other variables, the inter-group baseline differences were small and not statistically significant.

### The effect of training

**Fig 2:**
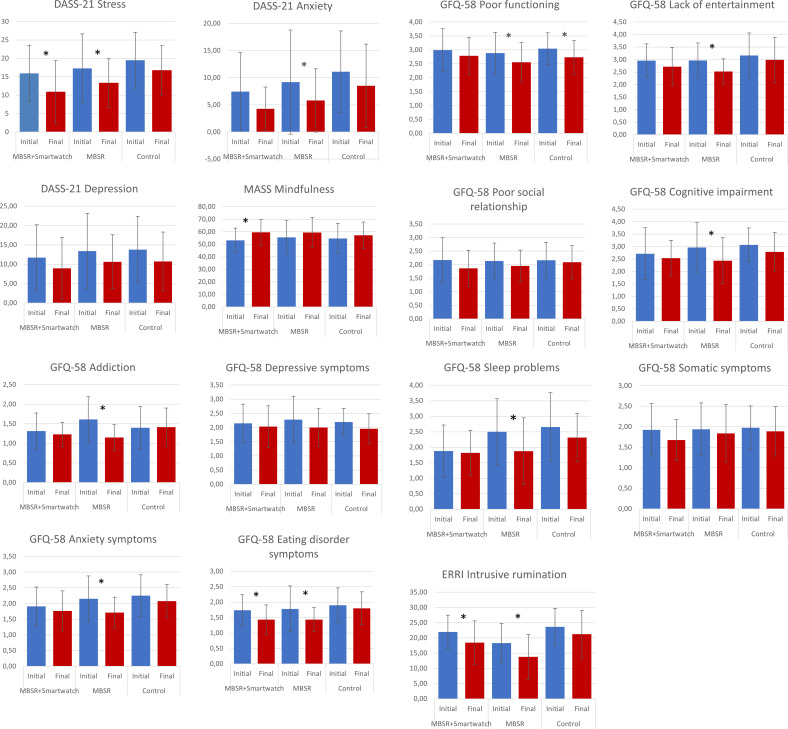
The mean scores from the initial and final measurements for all study groups. Statistically significant differences (*p* < 0.05) between each group’s initial and final measurements are marked with an asterisk (*). Error bars indicate standard deviations.

A change in the average level of several variables was observed in both MBSR participant groups after 8 weeks of practice, regardless of smartwatch usage (details are presented in Fig 2). Significant reductions in stress levels were observed in both MBSR participant groups, i.e., in the MBSR+smartwatch group (*Z* = -2.57, *p* = 0.01) and the MBSR group (*t*(18) = 2.15, *p* = 0.046). Additionally, these groups exhibited decreases in eating disorder symptoms, as measured by the GFQ-58, in both the MBSR+smartwatch group (*Z* = -2.06, *p* = 0.039) and the MBSR group (*Z* = -2.43, *p* = 0.015), as well as decreases in intrusive rumination, as measured by the ERRI, in both the MBSR+smartwatch group (*t*(22) = 2.10, *p* = 0.048) and the MBSR group (*t*(19) = 2.87, *p* = 0.01).

In the MBSR+smartwatch group, a specific significant increase in mindfulness (MASS) was observed after completing the MBSR training (*t*(22) = -3.24, *p* = 0.004). This effect did not occur in any other group.

In the MBSR group, different benefits of participating in the training were observed, which did not appear in individuals using a smartwatch nor in the control group. The results indicate a significant decrease in the severity of anxiety symptoms, as measured by DASS-A (*Z* = -1.97, *p* = 0.049) and GHQ-anxiety symptoms (*t*(19) = 2.52, *p* = 0.021). There was an improvement in overall functioning at work and outside of work, including GFQ-poor functioning (*t*(19) = 2.40, *p* = 0.027) and GFQ-lack of entertainment (*Z* = -2.59, *p* = 0.01), as well as a decrease in experienced cognitive difficulties - GFQ-cognitive impairments (*Z* = -2.40, *p* = 0.017), addictive tendencies - GFQ-addiction (*Z* = -2.85, *p* = 0.004), and sleep problems - GFQ-sleep problems (*Z* = -2.55, *p* = 0.011).

We also observed some changes among the participants of the control group - a significant decrease in GFQ-poor functioning (*t*(19) = 3.05, *p* = 0.007) appeared after 8 weeks of treatment. Details of all results are presented in Table 1.

## Discussion

We expected that both groups participating in the 8-week MBSR training would experience a reduction in stress levels and an improvement in well-being, with a stronger effect observed among participants who received additional support through interoceptive signal monitoring via a smartwatch. The results of our study showed that participants after the MBSR training without smartwatches (the MBSR group) experienced a decrease in stress levels (DASS-S) and anxiety symptoms (DASS-A, GFQ-anxiety). We also observed improvement in overall well-being (GFQ), including a decline in experienced cognitive difficulties, addictive tendencies, sleep problems, eating disorder symptoms, and general poor functioning at work and outside of work. Additionally, individuals in the MBSR group also reported a reduction in intrusive rumination (ERRI). It is worth emphasizing that some of these effects also occurred in the MBSR group supported by smartwatches, including reductions in stress levels, symptoms of eating disorders, and intrusive rumination. These observations, particularly the stress reduction, confirm the effectiveness of MBSR as an intervention designed for individuals experiencing difficult situations [41,43]. Consistent with our results, previous research has shown that MBSR helps reduce substance abuse [91], improve sleep quality [92,93], influence cognitive functioning [94], change eating behaviors [95,96], as well as overall functioning in many aspects of life [97]. MBSR has been associated with decreased rumination [98,99]. The consistent reduction in anxiety symptoms observed in our study, regardless of the questionnaire used, aligns with findings from other studies [100–103].

Although the MBSR training by its nature should influence mindful abilities, in our study this influence was quite limited. We observed a significant increase in the MAAS score after 8 weeks of practice only in a group equipped with smartwatches. It is worth noting, however, that a meta-analysis of intervention studies based on mindfulness indicated that half of these studies failed to demonstrate an increase in subjectively perceived mindfulness [104]. Importantly, the absence of a significant increase in mindfulness did not preclude the benefits of participating in MBSR training. Participants experienced numerous advantages, including improvements in their overall emotional functioning. The lack of the expected significant increase in mindfulness, as assessed by MASS, may partially result from the method used to measure this construct. Several challenges have been identified in the assessment of mindfulness. Firstly, there is no universally accepted definition of mindfulness [105,106]. Some questionnaires reduce this concept to one dimension, such as the MASS [27], while others evaluate it across several dimensions [107]. Single-dimensional measures have been criticized, as some researchers argue that mindfulness relates to multiple factors contributing to mindful functioning [107,108]. Secondly, concerns have been raised about the design of the MASS itself, particularly its reliance on reverse-scored questions. These questions primarily assess the absence of mindfulness or lapses of inattentiveness to the present moment as more readily identifiable by the general population [106]. The MASS scale itself includes the level of attention to, and awareness of, what is occurring in the present moment [27]. Despite those criticisms, the MASS remains one of the most commonly used mindfulness questionnaires, and meta-analysis did not show that this questionnaire differed from others in sensitivity to detecting changes in self-report mindfulness [104]. Furthermore, researchers have suggested that mindfulness may not be fully captured through self-report questionnaires [106]. In non-clinical samples, mindfulness training tends to produce only a small effect on self-reported mindfulness [109]. Studies involving active control groups also fail to show significant differences in mindfulness levels between those groups and participants undergoing mindfulness-based intervention [104]. As highlighted by researchers, a mechanism that plays a greater role in improving well-being may be more effective self-regulation [104,110].

We also failed to confirm the impact of MBSR on reducing depressive symptoms, despite this effect being reported in many other studies [111–114]. The observed reduction in stress levels and anxiety symptoms in the MBSR group, without a corresponding decrease in depressive symptoms, may result from differences in their characteristics. According to the theoretical framework underlying the DASS-21 scale, the depressive state is divergent from stress or anxiety, being primarily associated with an absence of positive affect symptoms, such as sadness, hopelessness, and an inability to experience pleasure, which can negatively impact self-esteem and sense of efficacy [83,115]. Therefore it can be assumed that this aspect of functioning was less influenced by the MBSR training. However, researchers have noted that the impact of MBSR on depressive symptoms is not always straightforward [116]. Different analyses show that the effect size for reducing depressive symptoms through MBSR is generally small [117]. Furthermore, MBSR yields better effects in patients with clinical diseases than in non-clinical populations [118], as well as where depressive symptoms are more severe [114,116].

Our research has shown that enhancing the self-regulation process through increased body awareness and interoceptive skills, facilitated by smartwatch monitoring of physiological signals, can yield both beneficial and adverse effects on functioning across various domains. The analysis revealed that integrating a smartwatch into MBSR neither hindered nor enhanced the stress-reducing effect (DASS-S) typically associated with MBSR. On the other hand, using a smartwatch strengthened participants’ mindfulness tendencies (MASS) more than training without a smartwatch as well as reduced eating disorder symptoms (GFQ) and intrusive rumination (ERRI). However, the necessity of continuous monitoring of parameters via the smartwatch limited the effects of MBSR in areas related to overall defined comfort at work and outside of work. It also weakened the effects of MBSR-induced reduction in anxiety, addictive tendencies, sleep problems, or experiencing cognitive difficulties.

Our research results demonstrate that the effect of stress reduction under the influence of MBSR is predominant [41,43,45,91,119–121]. This effect appeared in both groups, regardless of whether they received additional support in the form of extra information provided by the smartwatch. Our study showed that interoceptive signals related to stress levels and other body-generated signals, delivered via the smartwatch, enhance the effect of stress reduction observed under the influence of MBSR. By observing in real-time the impact of their behavior on reducing stress levels, participants who use the smartwatch could better leverage the skills acquired during MBSR, resulting in an even greater stress reduction. Since this effect was evident in the domain of stress but not in anxiety symptoms, it can be presumed that this information contributed to an increase in the ability to identify negative emotions, which are the core of stress [83–85]. Emotional control is one of the elements of self-regulation of emotion, and the belief in one’s ability to control emotions reduces stress [122]. In our study, the ability to use information provided by the smartwatch supported the process of self-observation and control of affective states, contributing to a reduction in subjectively experienced life stress.

The previously mentioned observation indicated that smartwatch users experienced a stress reduction after MBSR training, while changes in anxiety symptoms measured by DASS-21 and GFQ-58 were not significant. It allows us to assume that stress and anxiety are not completely identical phenomena. According to the concept of a tripartite structure of anxiety and depression [115] on which the DASS-21 scale [83] is based, the presence of negative affect is specific to stress [83–85]. Stress-tension in the DASS-21 includes difficulty relaxing, irritability, agitation, persistent tension, a low threshold for becoming upset or frustrated, and a tendency to overreact to stressful events. Stress is also related to chronic worry [123]. On the other hand, in these questionnaires anxiety is specifically defined by a physiological hyperarousal. The distinction described above indicates that the MBSR training efficiently reduces negative affect (stress level), as well as physiological hyperarousal (anxiety level), but for some reason, using smartwatches limits this effect only to the stress area. It is important to note that anxiety questionnaires used in this study assess perceived rather than actual physiological hyperarousal indicators. This kind of interoception could have been distorted by various psychological factors, such as expectations and attention to bodily states. Using a smartwatch to observe actual physiological parameters naturally reduces perceptual distortions and allows for a more accurate assessment of the physiological state. Therefore, it cannot be ruled out that participants in the non-smartwatch MBSR group overestimated the extent of their anxiety symptoms reduction after the training. Research indicates that physiological arousal does not necessarily correspond to a similar level of perceived stress [124]. A significant role is played by negative thought patterns or unhelpful beliefs [125–128], as well as personality traits such as high neuroticism [129–133]. It is also worth noting that anxiety can arise not only in response to an immediately affecting stimulus but also as a result of anticipating its occurrence. Therefore, the information provided by the smartwatch may have supported coping with the directly experienced stress, but not with the anticipated threat.

Analysis of the results showed that only three effects were consistent in both MBSR groups, regardless of whether they were equipped with a smartwatch or not. Besides the reduction of stress levels which was described earlier, we also observed the parallel reduction in perceived eating disorders (GFQ-58 score) and the reduction in intrusive ruminations (ERRI score). The first one is not surprising, because mindful eating practice is a component of the MBSR [39]. We hypothesize that the MBSR training may eliminate the emotion regulation strategy based on eating. Many studies have shown that using eating to cope with difficult emotions is usually inefficient [134–136]. The effectiveness of mindfulness has been previously demonstrated among individuals who use eating as an emotion regulation factor, as well as those suffering from eating disorders [137]. The possible shift in emotion regulation strategies, moving away from eating-based regulation, coupled with the improvement in emotion control associated with stress reduction, suggests that the MBSR training may enhance emotional regulation skills and that smartwatches with physiological parameters monitoring function can support this effect.

The other consistent effect observed in both MBSR groups was a reduction in the frequency of intrusive ruminations, which can be defined as a component of worry and stress reactions [98,99,123]. However, among smartwatch users, the change in this domain was not associated with a change in anxiety, which was primarily measured by perceived physiological symptoms such as hyperarousal and somatic tension. In the non-smartwatch MBSR group, the decrease in ruminations was associated with a reduction in anxiety. This difference in the specific patterns of change observed in response to the MBSR training in both groups can be explained by separating the concept of anxiety into anxious apprehension and anxious arousal [138–143]. Previous studies show that each of these parts of anxiety is driven by distinct biological mechanisms and is reflected in different patterns of brain activity [139]. Anxious apprehension is characterized by worry, negative repetitive thinking, and verbal rumination, encompassing the cognitive aspect, whereas anxious arousal refers to symptoms of physiological hyperarousal and somatic tension, involving the physiological or somatic aspect. The questionnaires used in our study to measure anxiety (the DASS-21 and GFQ-58 anxiety scales) were more focused on indicators aligned with the definition of anxious arousal, while the measurement of intrusive ruminations aligned with the definition of anxious apprehension. This interpretation is consistent with our earlier conclusion that the MBSR training effectively impacts the perceived affective and cognitive components of stress or worry. However, using smartwatches to observe actual physiological parameters strongly influences the perception of anxiety dimensions related to bodily sensations.

Prolonged use of a smartwatch facilitates the control of bodily signals important for self-regulation and well-being, yet simultaneously necessitates continuous engagement of attention. Therefore, the participants of the MBSR group using a smartwatch could experience both benefits and drawbacks. Continuously engaged attention contributed to increased awareness of these signals, as well as to stress reduction by gaining control over these signals and improving emotional regulation skills. However, this impeded improvement in overall psychological comfort. The improvements were manifested primarily in the affective domain (e.g., reduction in negative emotions specific for stress) and the cognitive domain (e.g., reduction in worry and intrusive ruminations), but without an impact on the perceived decrease in physiological arousal or somatic tension measured by the DASS scale. Given that effective self-regulation - encompassing the control of attention, emotions, thoughts, and behavior - is a key mechanism by which mindfulness improves mental health, our study suggests that both groups could enhance a different aspect of self-regulation. Smartwatch users could train one aspect of self-regulation, namely the ability to intentionally direct attention to signals from the body and information about experienced stress, and through an increased sense of control over these signals, effectively regulate their negative emotions. Improvements in various aspects of self-regulation among both groups participating in MBSR may have contributed to the observed changes. Smartwatch users, due to the necessity of directing attention to bodily signals to a greater extent than those who did not use a smartwatch, demonstrated enhanced mindfulness. However, in our study, the ability to regulate attention did not translate into behavioral regulation, particularly in areas such as workplace and non-work functioning, sleep quality, substance use, and adaptive forms of entertainment. Moreover, it has been shown that two aspects of interoception, namely interoceptive attention and interoceptive sensing, may differently moderate the relationship between smartwatch-derived information and perceived stress levels. On the one hand, awareness of stress levels enables individuals to implement appropriate strategies to reduce stress [51,67]. Directing attention can either amplify or diminish the experience of an ongoing physiological stress response, leading to an increase or decrease in perceived stress levels, respectively [144,145].

It appears that, despite their potential benefits in modifying health habits, smartwatches could be a distraction for mindfulness learners. The use of smartwatches may have resulted in greater involvement of one mechanism and suppression of others. The importance of smartwatches for stress management may not be as straightforward as it initially seems. For example, Pope et al. showed that wearing a smartwatch complementary to an educational intervention did not provide the expected additional benefits [146]. In this study, participants reported annoyance with wearing a smartwatch and the model’s limited functionality. The attitude towards the smartwatch hindered self-regulation based on observed parameters and did not contribute to achieving favorable changes. Similarly in a study by Smith et al. respondents who participated in mindfulness-based training using an app combined with monitoring of physiological parameters, mainly changes in breathing, had more significant decreases in stress levels and perceived anxiety after the intervention but no effect on improvements in global emotional experience [68]. In another research, it was found that the intrusiveness of sensors may even weaken the beneficial effect of stress intervention [147].

### Conclusions

In conclusion, despite the limitations imposed by the smartwatch on certain aspects of functioning, the strengthening effect of stress reduction observed in our study is noteworthy. Monitoring interoceptive signals by smartwatch may support the ability to recognize negative emotions and bodily signals associated with these emotions. This heightened awareness of emotions can contribute to improved control over negative emotions, resulting in reduced stress levels, and improved emotional regulation skills. Also worth noting is the increase in mindfulness among participants who monitored bodily signals using a smartwatch. The findings suggest that the use of smartwatches may enhance mindfulness toward bodily sensations, in our study to a degree even greater than participating in MBSR. However, increased awareness of these signals does not necessarily translate into improved well-being if it is not accompanied by non-worrying and acceptance of those sensations’ attitudes. In other words, just being aware of body signals is not sufficient. In this context, the training focused on understanding the relationship between physiological parameters and mental health could be beneficial. Excessive focus on bodily symptoms may exacerbate anxiety-related symptoms. For example, biased attention to bodily sensations and an over-sensitivity in interpreting these sensations is observed in people with emotion regulation difficulties [53]. Therefore, psychoeducation on interoception would be critical for fully unlocking the functionality of smartwatches. It is crucial to understand how users perceive and trust physiological stress data to design intuitive technologies that aid decision-making and well-being.

It is also important to highlight that the smartwatch model used in our study was preparing information on the probable stress level, which made it much easier to interpret and facilitated stress reduction. The application of signals from smartwatch for other aspects of emotional functioning proved to be more complex and possibly too challenging for improvements in these areas. In this context, it is worth mentioning that immediate feedback about the body, such as notifications of high stress level at a specific moment, can enhance the ability to recognize early signs of stress and identify the characteristics of situations that trigger it [148]. Real-time information may play a distinct role in developing interoceptive awareness compared to data presented as aggregated averages. The ability to utilize these signals to adapt the body to environmental demands is particularly significant. However, the literature highlights that devices monitoring physiological signals and presenting information as numerical values or graphs may fail to integrate with a coherent body image. This disconnect can lead to distortions in body perception and potentially impair self-awareness [149]. The results of our study also suggest that the simultaneous learning of MBSR techniques and enhancement of interoception through smartwatches may be overly demanding for participants.

It is worth noting that the results we observed may be distorted due to the fact that a large percentage of participants withdrew from the study before it began. This may have led to a specific selection of participants who were distinguished by some trait or type of motivation. Additionally, the dropout during the study did not occur evenly across groups. The highest number of dropouts was in the MBSR group. It can be assumed that participants who gained the least benefit from MBSR chose to withdraw. This fact may have influenced the observed larger change in the MBSR group.

Furthermore, individual differences between participants may have affected the observed results. We have tried to counter this in various ways. Potential differences in resting stress levels and other aspects of mental health were verified using appropriate statistical methods. We also applied strict inclusion criteria to increase homogeneity of groups. Nevertheless, the impact of personality traits on the results, especially on the observed differences in anxiety symptoms, remained not completely assessed.

Our results show, however, that additional support for MBSR training through the provision of physiological signals generated by the smartwatch, along with simple information about stress levels, may enhance the stress reduction effect. Furthermore, additional monitoring of bodily sensations could contribute to an increase in mindfulness. However, the smartwatch provided to individuals with high stress levels limits its utility in other areas of mental health, such as not contributing to the reduction of anxiety levels. Smartwatches can therefore support MBSR in stress reduction, but their impact on other mental health benefits of MBSR is limited.

### Limitations

The limitation of our study is its exclusive reliance on self-report measures, without incorporating an analysis of objective physiological data. Additionally, it is important to emphasize that our findings apply only to individuals experiencing high levels of stress. Due to the study’s eligibility criterion of elevated stress levels, our results should not be generalized to the broader population. Another limitation is the absence of a second control group that would have used smartwatches for 8 weeks without participating in the MBSR intervention. The inclusion of such a group would have allowed us to isolate the effects of monitoring interoceptive signals from smartwatches on mental health and to determine whether this alone could contribute to stress reduction in individuals experiencing high level of stress. Furthermore, our study did not assess subjective body awareness or interoceptive awareness. Incorporating these measures could help clarify whether these factors influence the observed changes among MBSR participants, both with and without smartwatch use.

Future research may benefit from integrating additional psychoeducational components to optimize smartwatch features for enhancing emotional regulation. Providing supplementary psychoeducation on the interplay between emotions, thoughts, behaviors, and physiological responses could facilitate the development of self-regulation skills and contribute to improved mental health outcomes. Additionally, the feedback generated by smartwatches regarding stress levels was more easily interpreted by users who had improved their stress management abilities. However, utilizing simple stress level notifications to enhance overall mental health may not be straightforward and could require additional support beyond the current functionalities of smart devices.

## Supporting information

S1 Dataset Scale scores of all participants. (XLSX)
